# Application of Plant Growth-Promoting Bacteria from Cape Verde to Increase Maize Tolerance to Salinity

**DOI:** 10.3390/antiox12020488

**Published:** 2023-02-15

**Authors:** Catarina Cruz, Paulo Cardoso, Jacinta Santos, Diana Matos, Carina Sá, Etelvina Figueira

**Affiliations:** 1 Department of Biology, University of Aveiro, 3810-193 Aveiro, Portugal; 2 Centre for Environmental and Marine Studies-CESAM and Department of Biology, University of Aveiro, 3810-193 Aveiro, Portugal

**Keywords:** maize, salinization, plant growth-promoting bacteria (PGPB), sustainability, arid regions

## Abstract

Salinity constitutes a major abiotic factor that negatively affects crop productivity. Inoculation with plant growth-promoting bacteria (PGPB) is proven to increase plant tolerance to abiotic stresses and enhance plant growth, development and productivity. The present study aims to increase the resilience of crops to salinity using bacteria from the microbiome of plants growing in saline environments. For that, the halotolerance of bacteria present in the roots of natural plants growing on Sal Island, which is characterized by its arid environment and maritime influence, was determined, with some strains having extreme halotolerance. Their ability to produce plant growth-promoting traits was evaluated, with most strains increasing indole acetic acid (26–418%), siderophore (>300%) and alginate (2–66%) production and phosphate solubilization (13–100%) under salt stress. The strains evidencing the best performance were inoculated in maize (*Zea mays* L.) plants and their influence on plant growth and biochemical status was evaluated. Results evidenced bacterial ability to especially increase proline (55–191%), whose osmotic, antioxidant and protein-protecting properties reduced protein damage in salt-stressed maize plants, evidencing the potential of PGPB to reduce the impact of salinity on crops. Enhanced nutrition, phytohormone production and osmolyte synthesis along with antioxidant response all contribute to increasing plant tolerance to salt stress.

## 1. Introduction

Salinization is spreading globally, being present in more than 100 countries and covering all continents [1]. Given climate change predictions, salt-affected areas are expected to increase in the future, due to the temperature and sea level rise, which will lead to increased evaporation, salinization and imbalance in groundwater systems [1,2,3,4]. Salinization can occur through natural processes, mainly occurring in arid and semi-arid areas and near the shoreline, but also anthropic activities [5]. Some human influences on soil salinity include inappropriate irrigation and drainage practices [6]. Soil salinization is often associated with sodification, the natural or anthropogenic accumulation of sodium in the soil, that is globally affecting about 210 million ha [5,7]. Recent data [1] show that over 45 million ha (25 to 30%) of irrigated land worldwide is affected by salinity. Irrigated land only represents 15% of the total land that can be cultivated, but it is responsible for the production of one-third of the world’s food [1,8,9], thus evidencing the impact that salinization can have on crop productivity and food security.

Soil salinization is responsible for reduced growth, yield and even crop failure in severe cases. It is proven that salinity leads to deficient water uptake by plants due to reduced osmotic potential, and Na^+^ ions may also cause ion toxicity effects and disturb plants’ nutritional balance [3]. Recent studies showed that exposure to salt adversely affects crucial stages of the plant growth cycle, such as seed germination, described in many species such as *Zea mays* [10] and *Triticum aestivum* [11], by altering protein metabolism, disturbing hormonal balance and reducing the use of seed reserves. Besides germination, salinity also negatively impacts major plant processes, such as photosynthesis, lipid metabolism and protein synthesis [9], and usually causes oxidative stress due to the generation of reactive oxygen species (ROS) [12]. The negative effects of ROS are usually associated with damage to cellular structures, such as membranes, but also with metabolic (protein denaturation), physiological (pigment breakdown) and DNA damage [13].

Plants can be classified either as glycophytes or halophytes, depending on their sensitivity to salinity and ability to grow in saline soils. Overall, most crops (including maize) belong to the first category, having a low tolerance to salinity [14]. Plants developed mechanisms to tolerate saline environments, such as morphological changes, compartmentalization or exclusion of toxic ions, biochemical adaptation, osmotic adjustment and hormonal regulation [13,14]. Under saline exposure, the increase in intracellular solute concentration (osmotic adjustment) maintains tissue turgidity and is critical for plant survival in saline conditions [14,15]. One of the most efficient and abundant compatible solutes is proline, whose synthesis can be dramatically enhanced by salt stress [16,17]. Besides osmotic adjustment, proline can also act as a reactive oxygen scavenger, a stabilizer of protein conformation and a protector of membrane integrity under salt stress [14,18,19]. Therefore, conditions that increase proline levels in plant cells will certainly contribute to increasing plant tolerance to salinity.

Presently, intensive research on the mitigation of salt stress effects on plants is being pursued with the aim to ensure food security by enhancing crop tolerance and productivity in salt-affected land. Plant roots interact with microorganisms such as bacteria, some of them promoting plant growth. These plant growth-promoting bacteria (PGPB) can have a direct effect through phosphate and potassium solubilization of phytohormones (such as gibberellins and auxins) synthesis, nitrogen fixation, siderophores and osmolyte production and/or by inducing systemic resistance and tolerance to stress [20,21,22,23,24]. Research on plant growth promotion by bacteria resulted in a high number of commercially available bacterial biofertilizers with different specificities. However, the vast majority of bioinoculants do not take into account abiotic factors such as drought and salinity that are expected to increase in the coming decades. Therefore, it is necessary to look for new solutions that maintain plant growth promoting efficiency in crops in stressful conditions. One of the strategies is the use of bacteria that are resilient to stressful environmental conditions and that simultaneously maintain a high plant growth-promoting ability. For this reason, bacteria have been isolated from harsh conditions across the globe. With the exception of North Africa, the bacterial diversity of the African continent, with its multiple habitats and extreme conditions has been explored little and can be an invaluable resource to produce next-generation biofertilizers.

Hereupon, this study aims at contributing to increasing the tolerance of crops to salinity using bacteria associated with the roots of plants growing in saline environments. Thus, bacteria were isolated from the roots of natural plants growing in an arid environment with maritime influence (Sal Island, Cape Verde). Bacterial isolates were identified, their halotolerance was determined and their ability to synthesize indole acetic acid (IAA) and siderophores to solubilize phosphate and to produce alginate was determined. The strains evidencing the best performance were inoculated in maize. The influence of salinity on *Z. mays* germination and early growth was assessed as well as the contribution of halotolerant bacteria to the mitigation of salinity effects on plants at biochemical and physiological levels. The comparison between salt-stressed and non-stressed plants allowed us to elucidate the bacterial contribution to stress alleviation.

## 2. Materials and Methods

### 2.1. Bacterial Strains

Plants (*Acacia albida* and *Amaranthus viridis*) sampled from Sal Island, Cape Verde (16°35′33″ N–22°55′29″ W) were used to isolate the bacterial strains. Eighteen strains prevailed after identification, and for each strain, an accession number was given in Table S1.

### 2.2. Bacterial Tolerance to Salinity

The method to determine the halotolerance of the bacterial strains was adapted from Cardoso et al. [25]. To assess the osmotolerance of the strains to salinity and to determine the concentration of NaCl that inhibits 50% of the bacterial growth (IC_50_), 100 μL of inoculum was added to 5 mL of modified yeast mannitol broth with increasing concentrations of NaCl (0, 2, 3, 4, 5, 6, 7 and 8%). The inoculated tubes were incubated for 15 h (until late logarithmic growth) in an orbital shaker (150 rpm) at 26 °C, and the optical density (OD) of each tube (at 620 nm) was recorded and used to calculate the IC_50_. Bacterial strains were grown again at the same temperature, agitation and incubation time, but only at NaCl IC_50_ and control (no supplementation with NaCl). For NaCl IC_50_ four replicates in two independent experiments were performed. For the control, values of NaCl and polyethylene glycol (PEG) experiments [26] were used, with averages calculated from eight replicates in four independent experiments.

After incubation OD was determined, tubes were centrifuged at 10,000 rpm for 10 min at 4 °C, and the supernatant was discarded. The pellet was stored at −20 °C for further biochemical analysis. The halotolerance of the bacterial strains was classified according to Cardoso et al. [25]: tolerant (2.1% < IC_50_ ≤ 3.6%), highly tolerant (3.6% < IC_50_ < 5.4%) and extremely tolerant (IC_50_ ≥ 5.4%).

### 2.3. Plant Growth Promoting Abilities

#### 2.3.1. Siderophore Production

The ability to produce siderophores was determined according to Arora and Verma [27]. Strains were inoculated in yeast mannitol broth medium containing 1 g/L of peptone (control) and with the NaCl IC_50_ concentration of each strain (three replicates each) and incubated at 150 rpm for 15 h. The OD was measured, cultures were centrifuged (12,000 rpm, 5 min, 4 °C) and the supernatant was collected. Samples and chrome azurol S reagent (CAS) in a ratio of 13:2 (*v*/*v*) were pipetted to a microplate and absorbance was measured at 630 and 750 nm after 20 min incubation. The siderophore production (percent siderophore unit–PSU) was quantified by the formula described by Arora and Verma (2017): ((Ar − As) × 100)/Ar), where Ar stands for the absorbance of the blank and As represents the absorbance of the samples. Results were expressed in percent siderophore units per optical density (PSU/OD).

#### 2.3.2. Alginate Production

To quantify alginate the method followed by Johnson et al. [28] was used. Each bacterial strain was inoculated, incubated and collected as mentioned before (Section 2.2). Three fractions of the total alginate (alginate in the medium, attached extracellularly to the bacterial cell wall and intracellular alginate) were obtained. 

To obtain the alginate present in the medium, after centrifugation of bacterial cultures in the same conditions described before (Section 2.2), 165 μL of the supernatant was transferred to a microtube, to which 35 μL of 517 mM ethylenediamine tetraacetic acid (EDTA) was added. The sample was incubated at 37 °C for 1 h and centrifuged (Section 2.2) and the supernatant was used to determine the alginate present in the medium.

Alginate attached to the cell wall was obtained by resuspending the pellet in 1 mL of dH_2_O, centrifuging (Section 2.2), and discarding the supernatant. Then 200 μL of 517 mM EDTA was added to the pellet, vortexed and incubated at 37 °C for 1 h. After incubation, the microtubes were centrifuged (Section 2.2) and the supernatant was used to determine the alginate extracellularly attached to the bacterial cell wall.

The pellet (intact cells) was resuspended in 300 μL of dH_2_O and lysed with an ultrasonic probe for 30 s at 0.6 Hz of amplitude, always keeping the samples in an ice bath to prevent overheating and centrifuged (Section 2.2). The supernatant was used to determine the alginate present in the medium. From the three factions, 100 μL of supernatant and 100 μL of 120 μM dimethylmethylene blue were pipetted into a 96-well microplate. After 10 min at room temperature, the absorbance was measured at 525 and 595 nm. A standard curve of alginate was used. Results in the three fractions were expressed in μg/mL/Optical Density.

#### 2.3.3. Indole Acetic Acid Production

The methodology of Gordon and Weber [29] with some alterations [30] was used to measure the production of indole acetic acid (IAA) in the presence (IC_50_) and absence of NaCl. Each bacterial strain was inoculated, incubated and collected as mentioned in the sections before.

In a 96-well microplate, 100 μL of supernatant was reacted with 200 μL of Salkowsky reagent for 10 min at room temperature. The absorbance was measured at 530 nm. IAA concentration was determined using IAA (Sigma, St. Louis, MO, USA) as a standard. Data are presented as the concentration of IAA (μg/mL) normalized to the OD of each strain.

#### 2.3.4. Phosphate Solubilization

The bacteria’s ability to solubilize phosphate was determined using the method of Nautiyal [31], with modifications that allowed to determine the ability to solubilize phosphate under salt stress. Bacteria were grown in modified Pikosvakaya medium, composed of 10 g of D-glucose, 5 g of tricalcium phosphate (Ca_3_(PO_4_)_2_) (Sigma), 0.5 g of ammonium sulfate ((NH_4_)_2_SO_4_) (Sigma), 0.2 g of NaCl (Sigma), 0.1 g of magnesium sulfate heptahydrate (MgSO_4_·7H_2_O) (Merck, Rahway, NJ, USA), 0.2 g of potassium chloride (KCl) (Merck), 0.5 g yeast extract (Alfa Aesar, Haverhill, MA, USA), 0.002 g of manganese sulfate monohydrate (MnSO_4_·H_2_O) (Sigma) and 0.002 g of iron (II) sulfate heptahydrate (FeSO_4_·7H_2_O) (Merck). The modified medium was sterilized in an autoclave for 20 min, at 120 °C. The bacteria were inoculated, and the plates were incubated for 10 days at 26 °C. After 10 days the colony and halo zone diameter (including the colony) were measured, allowing us to calculate the solubilization index (SI = Halo diameter/Colony diameter).

### 2.4. Plant Experiments

#### 2.4.1. *Zea mays* Tolerance to NaCl

*Z. mays* seeds used were certified trial seeds from Pioneer Optimum AQUAmax hybrids, variety P9911. 

Seeds were germinated as described by Cruz et al. [26]. Cups were watered with solutions differing in NaCl concentration (0 to 15%), initially with 40 mL and after with 10 mL whenever the surface sand was dry. Three replicates per concentration were performed.

Plants were grown in the same conditions as described by Cruz et al. [26]. After 7 days of growth, plants were collected. The root and shoot height and fresh weight were determined. To determine the dry weight, the plants were dried (60 °C) until a constant weight. The NaCl concentration closer to IC_50_ was 1% and thus this concentration was used in subsequent work. 

#### 2.4.2. Greenhouse Experiment

*Z. mays* seeds were hydrated in the same way as mentioned before and after germination were sown in cups with river sand. Two conditions (0% and 1% NaCl), and three replicates each with three seeds per cup were conducted. Plants were inoculated with the 18 bacterial strains and a control (non-inoculated plants) was also included. Since the exposure of the plants to NaCl was carried out simultaneously with the exposure to polyethileneglicol-6000 (PEG) [26] the control plants were the same for both experiments.

Plants were grown in the same conditions as mentioned before. However, initially, cups were watered with 50 mL of dH_2_O and 1% NaCl and after 2 more days with 15 mL. Morphometric parameters of shoots and roots were determined. The length and weight of the roots and shoots of each plant were measured and weighed, respectively. Shoots and roots were either promptly used to quantify photosynthetic pigments or frozen (−20 °C) for further use. Biochemical analysis was performed in plants inoculated with bacterial strains (A, D, F, G, Q, R, S and T) promoting seedlings in comparison to the control. 

#### 2.4.3. Photosynthetic Pigments 

Chlorophylls and carotenoids were extracted in the dark with a mortar and pestle using 2 mL of cold acetone (80%) and quantified as described in Cruz et al. [26]. The homogenate was pipetted into a microtube and centrifuged at 10,000 *g*, for 20 min at 4 °C. In a 96-well microplate 150 μL of sample supernatant and 150 μL of acetone (80%), were added; the blank was 300 μL of 80% acetone. 

### 2.5. Biochemical Analysis

#### 2.5.1. Extraction 

The pellet obtained by centrifugation of bacterial strains grown at the control and NaCl IC_50_ conditions (Section 2.2) was resuspended in sodium phosphate buffer and extracted as described in Cruz et al. [26]. According to the OD of each sample, a volume (between 500µL and 1500 µL) of the buffer was added. Bacterial cells were lysed with an ultrasonic probe for 30 s at 0.6 Hz, always keeping the samples in an ice bath to prevent overheating, and centrifuged at 10,000× *g* for 10 min at 4 °C. Both fractions were used immediately or frozen at −20 °C until used.

Plant shoots (0.2 g frozen material) grown in two salt conditions (0 and 1% NaCl) and inoculated or not with bacterial strains (2.4.2) were extracted with potassium phosphate buffer [32] and centrifuged at 10,000 rpm for 10 min at 4 °C. The supernatant and pellet were used immediately or frozen (−20 °C) until used. 

For proline, pelleted bacterial cells were suspended in 3% sulfosalicylic acid, lysed with an ultrasonic probe and centrifuged as mentioned before. The supernatant was collected and used immediately or frozen at −20 °C until further use. In plant shoots, 0.2 g of frozen material was homogenized with 0.5 mL of 3% sulfosalicylic acid (1:2 *w*/*v*) and centrifuged at 10,000 rpm for 10 min at 4 °C. The supernatant was collected and frozen (−20 °C) until further use.

#### 2.5.2. Lipid Peroxidation (LPO)

LPO was measured according to the method of Buege and Aust [33] based on the reaction of lipid peroxidation products such as malondialdehyde (MDA) with 2-thiobarbituric acid (TBA), forming TBARS (thiobarbituric acid reactive substances); 0.5% TBA (thiobarbituric acid) and 10% TCA (trichloroacetic acid) were added to the sample in an 8:1 (*v*/*v*) ratio. After incubation at 96 °C for 25 min, samples were measured at 532 nm. 

#### 2.5.3. Superoxide Dismutase (SOD)

SOD activity was determined using the method by Beauchamp and Fridovich [34]. The reaction was performed by mixing the sample with reaction buffer (50 mM Tris-HCl, pH 8.0, 0.1 mM diethylenetriaminepentaacetic acid—DTPA, 0.1 mM hypoxanthine, and 4 μM nitroblue tetrazolium—NBT) and xanthine oxidase (51.6 mU/mL) in a 2:9:1 (*v*/*v*/*v*) ratio. After incubation for 20 min, the absorbance was read at 560 nm. 

#### 2.5.4. Catalase (CAT)

CAT activity was determined using the methodology by Johansson and Borg [35]. In a 96-well microplate 25 μL of the sample was added (Section 2.5.1), followed by 125 μL of the reaction buffer (1M K_2_HPO_4_ and 1M KH_2_PO_4_, pH 7.0), 37.5 μL of methanol and 25 μL of 35.28 mM hydrogen peroxide (H_2_O_2_). After a 20 min incubation at room temperature, 37.5 μL of 10 M potassium hydroxide (KOH) and 37.5 μL of 34.2 mM purpald were added, followed by 10 min incubation at room temperature, and 12.5 μL of potassium periodate (KIO_4_). After an incubation of 5 min, the absorbance was read at 540 nm. Catalase activity was determined using a standard curve of formaldehyde.

#### 2.5.5. Glutathione S-Transferases (GST)

The activity of GST was determined according to Habig et al. [36]. The reaction buffer (100 mM potassium phosphate buffer, pH 6.5, 10 mM reduced glutathione, and 60 mM 1-chloro-2,4-dinitrobenzene was added to the sample in a 2:1 (*v*/*v*) ratio. The reaction was followed during 20 min, with 15 s intervals, at 340 nm. The activity of GST was determined using the extinction coefficient ε = 9.6 mM^−1^ cm^−1^ of the glutathionyl-dinitrobenzene (GS-DNB) conjugate. 

#### 2.5.6. Protein

Protein was determined according to Robinson and Hogden [37]. Biuret reaction solution was added to the sample in a 12:1 (*v*/*v*) ratio and incubated for 10 min. Samples were read at 540 nm, and bovine serum albumin (BSA) was used as a standard. 

#### 2.5.7. Protein Carbonylation

The determination of protein carbonyl groups was performed as described in Mesquita et al. [38] and Udenigwe et al. [39]. The sample was mixed with 10 mM DNPH at a 1:1 (*v*/*v*) ratio and incubated for 10 min at room temperature. After, 6 M NaOH was added to the mixture in a 1:4 (*v*/*v*) ratio and incubated for 10 min. Absorbance was measured at 450 nm. 

#### 2.5.8. ETS

ETS activity was determined in plants according to King and Packard [40]. In a 96-well microplate 35.7 μL of supernatant, 107 μL of Tris buffer (0.13 M Tris-HCl and 0.3% Triton X-100, pH 8.5), 35.7 μL of NAD(P)H and 71.4 μL of 8 mM p-IodoNitroTetrazolium (INT) was added. The absorbance was read at 490 nm for 15 min in 25 sec intervals. 

#### 2.5.9. Soluble Sugars

Soluble sugars were determined in plants following Dubois et al. [41]. In a microtube was added 10 μL of supernatant, 100 μL of 5% Phenol and 600 μL of 98% Sulfuric acid (H_2_SO4) and well mixed. After a 30 min incubation at room temperature, 300 μL from each microtube were transferred to a 96-well microplate and the absorbance was read at 492 nm. 

#### 2.5.10. Proline

Proline was determined following the method described by Bates et al. [42]. To 50 μL of the sample 50 μL of acid ninhydrin and 50 μL of glacial acetic acid were added. The microtubes were pierced and incubated for 1 h at 100 °C; the reaction was stopped by placing the samples in ice, after cooling, 125 μL of the samples were added to a 96-well microplate. The absorbance was measured at 520 nm and proline standards were used. 

### 2.6. Statistical Analysis

Hypothesis testing was performed by Permutation Multivariate Analysis of Variance (PERMANOVA) [43], using PRIMER version 6.1.16 for Windows. Salinity conditions (Control and IC_50_) were used as fixed factors. 

Significant differences were considered for *p* ≤ 0.05 and were identified in figures with single asterisks (for *p* ≤ 0.05) and double asterisks (for *p* ≤ 0.01). 

A Principal Coordinates Ordination (PCO) was made similarly to Cruz et al. [26] but substituting drought stress with salt stress. The Pearson correlation values are expressed in Table S6.

## 3. Results

### 3.1. Characterization of Strains and PGP Traits

**Figure 1:**
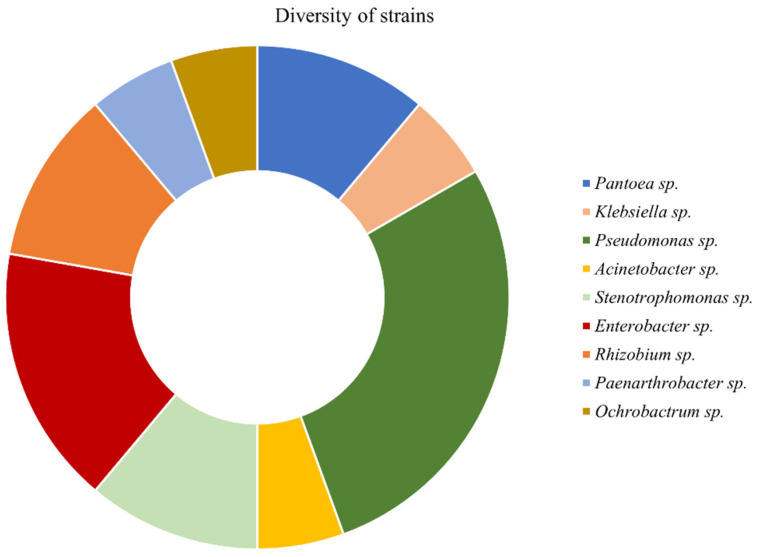
Bacterial genera of strains isolated from two natural plant species growing in Sal Island (Cape Verde) soils. For more information, related to bacterial strains see Supplementary Table S1.

The 18 strains isolated from two natural plant species growing in Sal Island (Cape Verde) belonged to nine genera (Figure 1). *Pseudomonas* was the most common genus (seven strains), followed by *Enterobacter* (three strains) and *Rhizobium*, *Stenotrophomonas* and *Pantoea* genera (two strains). The other genera (*Klebsiella*, *Acinetobacter*, *Paenarthrobacter* and *Ochrobactrum*) were only represented by one strain.

**Figure 2:**
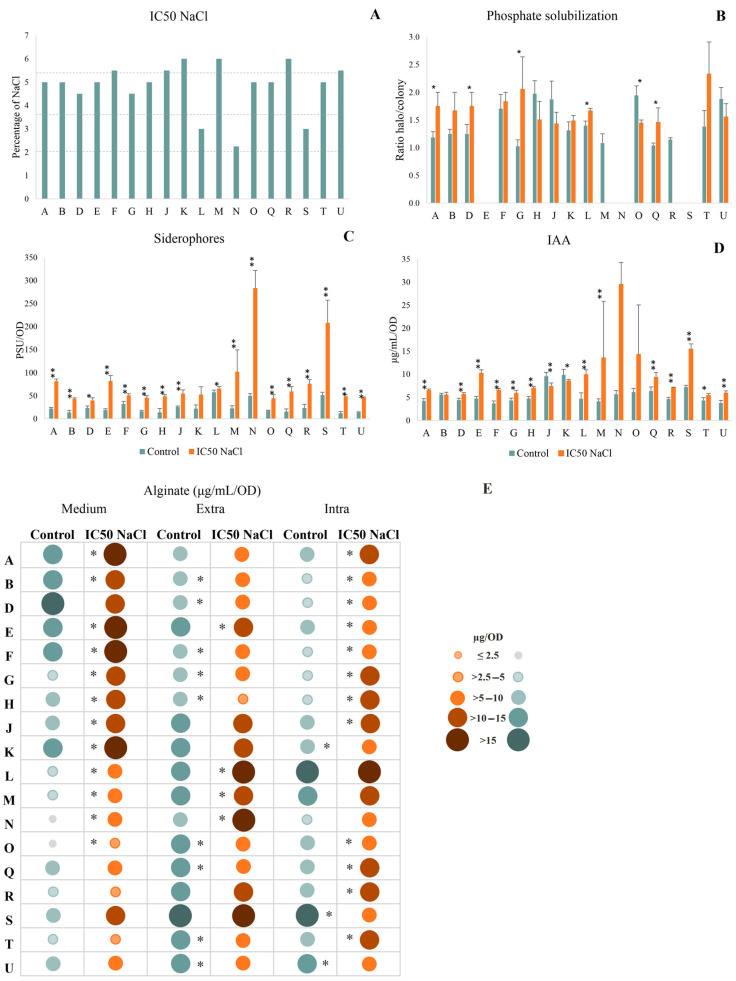
Effect of salinity on bacterial ability to produce PGP traits: (**A**) Percentage of NaCl that inhibits 50% bacterial growth (IC_50_ NaCl); (**B**) Phosphate solubilization, expressed by the ratio between halo and colony diameter; (**C**) Siderophore production, expressed in percent siderophore units per optical density (PSU/DO); (**D**) Indole acetic acid (IAA), expressed in μg/mL/OD and (**E**) alginate distribution in three fractions (medium, attached extracellularly to the cell wall–Extra and intracellular-Intra), expressed in μg/mL/DO. Bacterial strains: *Pantoea* sp. (A); *Klebsiella* sp. (B); *Pseudomonas* sp. (D); *Pseudomonas* sp. (E); *Acinetobacter* sp. (F); *Stenotrophomonas* sp. (G); *Enterobacter* sp. (H); *Enterobacter* sp. (J); *Pantoea* sp. (K); *Pseudomonas* sp. (L); *Rhizobium* sp. (M); *Paenarthrobacter* sp. (N); *Ochrobactrum* sp. (O); *Pseudomonas* sp. (Q); *Rhizobium* sp. (R); *Stenotrophomonas* sp. (S); *Pseudomonas* sp. (T); *Enterobacter* sp. (U). Values are means of three replicates + standard deviation. Significant differences compared to control (non-inoculated and non-salt exposed plants) were marked with single asterisks (*p* < 0.05) or double asterisks (*p* < 0.01). For means, standard deviation and statistical significance see Supplementary Table S2.

In Figure 2A three strains were classified as halotolerant (L, N and S), half of the strains (9) as highly halotolerant (A, B, D, E, G, H, O, Q, and T), with the remaining six strains classified as extremely halotolerant (F, J, K, M, R and U).

Phosphate solubilization was observed in 15 strains (Figure 2B), all characterized as moderate solubilizers, with the remaining three (E, N and S) not having the ability to solubilize phosphate. When exposed to salinity, 50% of the strains increased phosphate solubilization, significantly in A, D, G, L and Q, two strains (M and R) lost the ability to solubilize phosphate and in four strains salinity reduced this ability, significantly in strain O. Strains G and T become high P solubilizers under salt stress (ratio ≥ 2.0).

Under control and stress conditions, all strains are able to produce siderophores (Figure 2C). The production significantly increases in all bacteria exposed to salinity, with differences between stress and control higher than 300%. 

Under control conditions, all strains are able to produce IAA (Figure 2D), with J and K producing higher amounts of this phytohormone (>9 μg/mL/OD). Exposure to salt stress resulted in higher IAA production in 15 strains, significantly in 13 of them (A, D, E, F, G, H, L, M, Q, R, S, T and U). 

Alginate production in the medium, attached extracellularly to the cell wall and intracellularly can be depicted in Figure 2E. Overall, most strains increased alginate in the medium when exposed to salinity, significantly in 12 out of the 16 strains. Strains A, E, F and K showed higher alginate production in the medium, both in control and stress conditions. Exposure to salinity decreased extracellular alginate in 13 strains, significantly in nine of them. On the other hand, strains E, L, M and N increased extracellular alginate significantly under salt stress. Strain S stood out by producing a high amount of alginate extracellularly under control (18.29 μg/mL/OD) and stress conditions (18.72 μg/mL/OD). Overall, the extracellular alginate was higher than in the medium or intracellularly. Intracellular alginate had the lowest values of the three fractions. Overall, salinity increased intracellular alginate, with 14 strains increasing the production, significantly in 12 of them. 

### 3.2. Biochemical Changes from Bacterial Exposure to NaCl

#### 3.2.1. Cellular Damage

**Figure 3:**
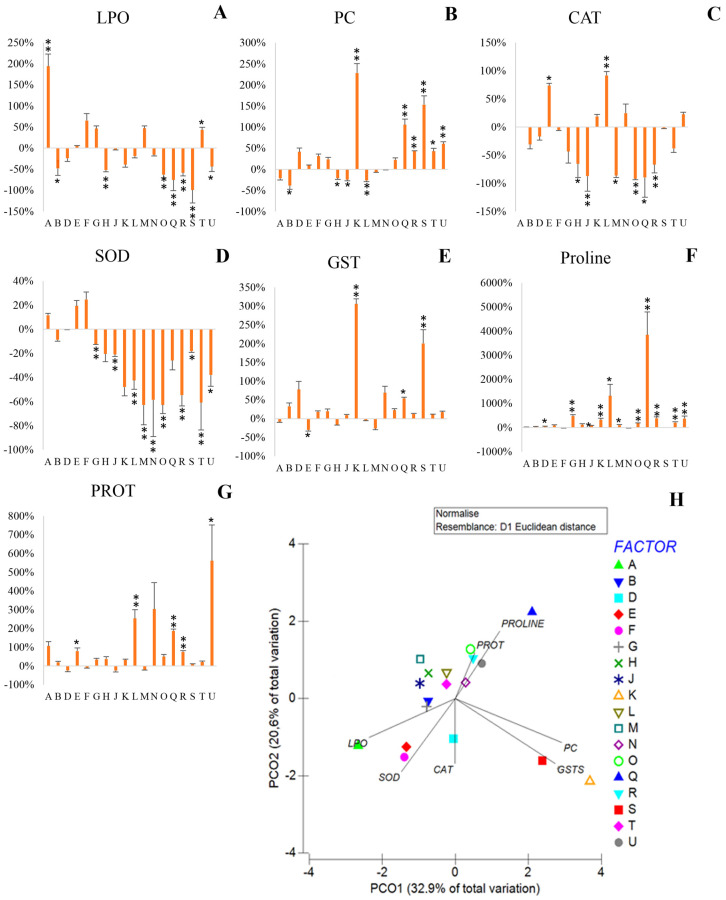
Effect of salinity on bacterial strains biochemistry: (**A**) Lipid peroxidation, (**B**) Protein carbonylation, (**C**) Catalase, (**D**) Superoxide dismutase, (**E**) Glutathione S-Transferase, (**F**) Proline, (**G**) Protein, (**H**) principal coordinates ordination of biochemical parameters under saline stress. Biochemical markers variation of bacterial cells exposed to IC_50_ NaCl relatively to control (not exposed to NaCl) are superimposed. Bacterial strains: *Pantoea* sp. (A); *Klebsiella* sp. (B); *Pseudomonas* sp. (D); *Pseudomonas* sp. (E); *Acinetobacter sp.* (F); *Stenotrophomonas* sp. (G); *Enterobacter* sp. (H); *Enterobacter* sp. (J); *Pantoea* sp. (K); *Pseudomonas* sp. (L); *Rhizobium* sp. (M); *Paenarthrobacter* sp. (N); *Ochrobactrum* sp. (O); *Pseudomonas* sp. (Q); *Rhizobium* sp. (R); *Stenotrophomonas* sp. (S); *Pseudomonas* sp. (T); *Enterobacter* sp. (U). Values are means of three replicates. Significant differences compared to control (non-inoculated and non-salt exposed plants) were marked with single asterisks (*p* < 0.05) or double asterisks (*p* < 0.01). Means, standard deviation and statistical significance are included in Supplementary Table S3.

When exposed to salt stress, the majority of the strains (12) showed lower lipid peroxidation (Figure 3A), significantly in seven of them (B, M, O, Q, R, S and U). However, some strains increased LPO under salinity (A, E, F, G, M and T), significantly in A and T. 

Overall, salinity increased protein carbonylation (Figure 3B), significantly in K, Q, R, S, T and U. On the other hand, strains B, H, J and L were able to significantly decrease protein damage under stress.

#### 3.2.2. Antioxidant Response

When exposed to salt stress, 45% of the strains reduced catalase activity by over 15% compared to the control (Figure 3C), significantly in H, J, M, O, Q and R strains. However, in five strains catalase activity (E, K, L, N and U) increased in salt stress, significantly in E and L, (3.2 and 2.5 µU per million cells, respectively).

Overall, the presence of salt stress negatively influenced SOD activity (Figure 3D), with only three strains being able to increase SOD activity (A, E and F), although not significantly. In fact, decreases of over 15% in 12 of the bacterial strains were observed, significantly in G, J, L, M, N, O, R, S, T and U. 

Exposure to salinity increased glutathione-S-transferase activity in 13 strains, significantly in K, Q and S (Figure 3E), with K and S registering higher values (11.11 and 10.16 µU/million cells, respectively). Although E showed a significant reduction in GST activity in salt stress, this strain displayed similar absolute values relative to the remaining strains under stress.

#### 3.2.3. Osmolyte Production-Proline

Salt stress increased proline production in all strains, significantly in 11 of them (D, F, J, K, L, M, O, Q, R, T and U) (Figure 3F). Significant increases in proline production over 1000% were recorded in strains L and Q, with respective contents of 4.97 and 16.26 µg of proline per million cells.

#### 3.2.4. Protein Content

Under stress, protein content increased by over 15% in 13 of the strains, significantly in E, L, Q, R and U (Figure 3G), with protein content ranging from 0.25 (R) to 1.07 (L) µg protein/million cells.

#### 3.2.5. Multivariate Analysis

A PCO was performed with the difference between salinity and control conditions, showing the effect of salt stress on bacteria. In the multivariate analysis (Figure 2H) it is possible to observe that PCO1 explains most of the salinity effects on bacterial biochemistry. The alterations from exposure to IC_50_ NaCl were highly correlated to protein damage and the antioxidant activity of GST, with Pearson correlations higher than 0.9. In the negative axis of PCO1 and positive axis of PCO2 bacterial strains less affected by salinity are placed; strains H, J, L, M and T were positioned in this quartile. In the negative axes of PCO1 and PCO2 are positioned strains with high membrane damage (A), high SOD (E and F) and CAT (D) activity. Strains K and S are positioned in PCO1 positive axis and PCO2 negative axis, showing higher PC and GST activity caused by salinity. On the positive side of the PCO1 and PCO2 axes N, O, R and U strains correlating with increased protein content and decreased SOD activity and lipid peroxidation content are positioned. Strain Q is isolated in this quartile and stands out by its high increase in proline content in salt stress.

### 3.3. Response of Plants to Salinity

#### 3.3.1. Biometric Parameters

**Figure 4:**
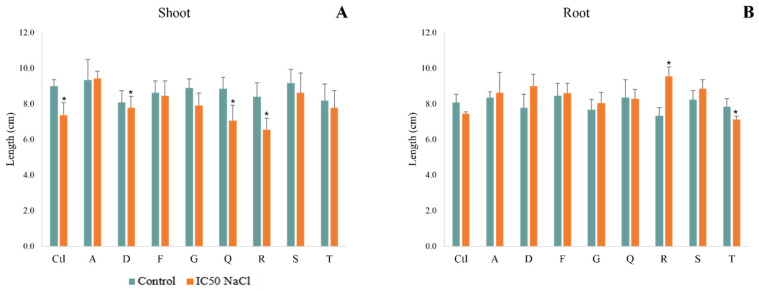
Maize plants grown for 7 days in non-saline and saline (1% NaCl) conditions. Biometric parameters: (**A**) fresh weight and (**B**) length of shoots; Non-stressed (greenish bars) and salt-stressed (orange bars) plants were inoculated with different strains: *Pantoea sp.* (A); *Pseudomonas* sp. (D); *Acinetobacter sp*. (F); *Stenotrophomonas* sp. (G); *Pseudomonas* sp. (Q); *Rhizobium* sp. (R); *Stenotrophomonas* sp. (S); *Pseudomonas* sp. (T). Values are means of three replicates + standard deviation. Significant differences compared to control (non-inoculated and non-salt exposed plants) were marked with single asterisks (*p* < 0.05). For means, standard deviation and statistical significance see Supplementary Table S4.

In non-saline conditions, the effect of bacterial inoculation on plant growth and biomass was mild with increases lower than 10% compared to non-inoculated plants. According to Figure 3A, it is possible to observe that exposure to salinity decreased shoot length in most plants, significantly in non-inoculated plants and in plants inoculated with D, Q and R (Figure 4A,B). Strains A, F and S promoted shoot length by more than 10% in plants exposed to salinity, with strain A significantly increasing (28%) this parameter compared to salt-stressed non-inoculated plants. When exposed to salinity, strains D, F, R and S significantly promoted root growth compared to salt-stressed non-inoculated plants, with increases of 21%, 15%, 29% and 19%, respectively (Figure 4B). 

None of the bacterial strains were able to significantly promote the fresh weight of shoots and roots, although some increments were recorded (Supplementary Table S2). Under salt stress, shoot fresh weight increased by 14% in plants inoculated with F, but not significantly. On the other hand, only strain D did not promote root FW by over 10%, but none of the remaining strains significantly increased root fresh weight compared to the control. Since fresh weight results were not significant, values are only expressed in the Supplementary Material (Table S4).

#### 3.3.2. Photosynthetic Pigments

**Figure 5:**
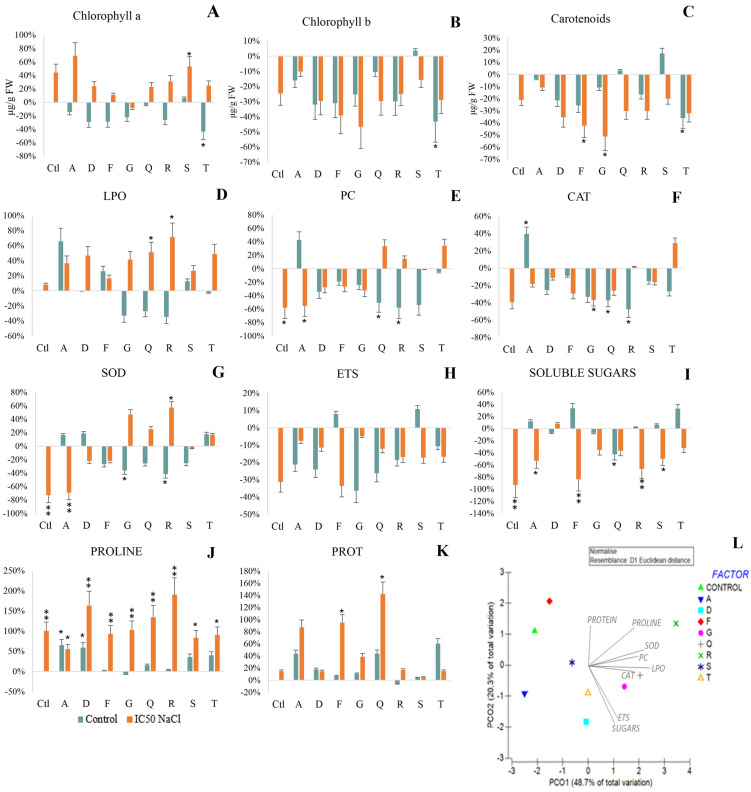
Maize plants grown for 7 days in non-saline and saline (1% NaCl-IC_50_) conditions. Variation of photosynthetic pigments and biochemical parameters relatively to control (non-inoculated and non-salt exposed plants): (**A**) chlorophyll a; (**B**) chlorophyll b; (**C**) carotenoids; (**D**) Lipid peroxidation; (**E**) Protein carbonylation; (**F**) Catalase; (**G**) Superoxide Dismutase; (**H**) Electron transport system; (**I**) Soluble sugars; (**J**) Proline and (**K**) Protein. (**L**) Principal coordinates ordination of biochemical parameters variation of salt-stressed relatively non-salt exposed plants. Non-stressed (greenish bars) and salt-stressed (orange bars) plants were inoculated with different strains: *Pantoea* sp. (A); *Pseudomonas* sp. (D); *Acinetobacter* sp. (F); *Stenotrophomonas* sp. (G); *Pseudomonas* sp. (Q); *Rhizobium* sp. (R); *Stenotrophomonas* sp. (S); *Pseudomonas* sp. (T). Values are means of three replicates. Significant differences compared to control (non-inoculated and non-salt exposed plants) were marked with single asterisks (*p* < 0.05) or double asterisks (*p* < 0.01). For means, standard deviation and statistical significance see Supplementary Tables S4 and S5.

In control conditions, only strain S promoted photosynthetic pigment synthesis, although not significantly (Figure 5A–C), and inoculation with the remaining bacterial strains decreased it, significantly in T. In salt stress, the application of PGPB significantly increased chlorophyll a production (A and S). On the other hand, exposure to salinity decreased chlorophyll b (Figure 5B) and carotenoid content (Figure 5C), significantly in the T strain for chlorophyll b and carotenoids in non-saline conditions, and in G and F strains for carotenoids in salt stress conditions.

#### 3.3.3. Cellular Damage

Under salt stress plants displayed higher LPO compared to non-stressed plants, significantly in plants inoculated with Q and R (Figure 5D).

Under non-saline conditions, inoculation reduced protein carbonylation (Figure 5E), significantly in Q, R and S strains. When exposed to salinity, PC significantly decreased in non-inoculated plants, and in those inoculated with A, but increased protein carbonylation in plants inoculated with Q, R and S, although not significantly. Strains D, F and G stood out by inducing lower protein carbonylation in plants both in non-saline and saline conditions.

#### 3.3.4. Antioxidant Response

In non-saline conditions, inoculation with strain A significantly increased CAT activity (Figure 5F), while inoculation with the remaining strains decreased it, significantly in Q and R. When exposed to salinity, CAT activity significantly decreased in plants inoculated with G. 

Under salt stress, SOD activity was significantly reduced in non-inoculated plants and in plants inoculated with A. On the other hand, inoculation with F, Q, R and T increased SOD activity under salt stress, significantly in R (Figure 5G), with values ranging from 0.22 (F) to 0.45 (R) U SOD/g FW.

#### 3.3.5. Metabolism

ETS activity was not significantly influenced by salinity or inoculation (Figure 5H). 

In non-stressed plants, almost all strains promoted protein synthesis (Figure 5K). Salt stress inoculation also increased protein levels, significantly in F and Q strains.

#### 3.3.6. Osmolyte Production

In non-saline conditions, inoculation with A, F, R, S and T promoted soluble sugars accumulation up to 30% (F and T), although not significantly (Figure 5I). On the other hand, a significant decrease in soluble sugars (SS) was observed in plants inoculated with Q. Overall, exposure to salinity negatively influenced the content of SS, significantly decreasing SS content in non-inoculated plants, and in plants inoculated with F, R and S strains. 

In non-saline conditions, most strains promoted proline accumulation relative to non-inoculated plants (Figure 5J), significantly in strains A and D. Under salt stress, proline content significantly increased in all inoculated plants (55–191%) compared to non-inoculated non-stressed plants, with content of over 100 mg of proline per g of FW. 

#### 3.3.7. Multivariate Analysis

The PCO (Figure 5L) shows the effect of bacterial inoculation on the response of plants to salt stress compared to plants inoculated with the same strain but non-exposed to NaCl. All biochemical parameters have correlations higher than 0.5, with SOD and proline having the highest correlations of 0.93 and 0.89, respectively. Salinity effects on non-inoculated plants and plants inoculated with F are positioned on the negative part of the PCO1 axis and the positive side of PCO2, showing the negative impact of salinity on the electron transport system and soluble sugar content. Being nearest the origin, plants inoculated with strain S were affected less by salinity. The response of plants inoculated with the R condition is positioned on the positive side of both axes, evidencing a strong correlation with proline content, but also with LPO and SOD activity. Strains G and Q are both positioned on the positive side of PCO1 and near the origin of PCO2 correlating strongly with increases in membrane damage (LPO) and CAT activity. Plants inoculated with strains, D or T, are positioned on the negative side of axis PCO2 and near the origin of PCO1, correlating strongly with ETS and soluble sugars. Lastly, the condition where plants were inoculated with strain A is positioned on the negative side of both axes evidencing the low impact of salinity on proline content and the sharp decrease in SOD activity and protein carbonylation.

## 4. Discussion

The results obtained allowed us to fulfill the proposed objectives, which were to clarify the effect of salt stress on bacterial strains isolated from an arid natural habitat with a strong maritime influence and on maize plants inoculated with these bacteria. The biochemical mechanisms underlying the tolerance of both plants and bacteria to salinity were also performed and allowed us to understand the mechanisms bacteria resort to, to tolerate salt stress and the influence that inoculation with these bacteria has on the response of plants to salinity. 

Some studies already described the positive influence of inoculation with PGPB in *Z. mays* under salt stress [44,45,46] and the application of halotolerant bacteria as a more efficient option to enhance plant growth under salt stress [47,48,49]. However, the biochemical mechanisms underlying the halotolerance of bacteria and the increase in plant tolerance to salt granted by inoculation with these bacteria remain poorly studied. 

The results obtained in the present study showed that all strains were halotolerant, with nine strains being characterized as highly halotolerant, and six strains as extremely halotolerant. Plant growth-promoting traits evaluated showed that all strains were capable of producing IAA, siderophores and alginate and that in most bacteria salt stress increased these abilities, elucidating contradictory information already published. IAA stimulates cell proliferation and growth under saline conditions and improves nutrient acquisition [50], with its production being broadly studied in halotolerant PGPB [48,51,52]. There are bacterial genera recognized for producing IAA under salt stress, such as *Pseudomonas*, and *Stenotrophomonas* [52], and it is assumed that the ability to synthesize IAA is a common PGP trait of halotolerant bacteria [50]. However, in some studies, it was concluded that exposure to salinity decreased the production of IAA [53], even when the strains were considered halotolerant [51]. Results from this study evidenced the bacterial ability to produce IAA in control and stress conditions, with IAA synthesis increasing in most strains under salt stress, significantly in 72% of them. This result is relevant since the higher growth of the root will allow exploring a higher volume of soil, and therefore, more access to nutrients such as K^+^, which plays an important role in osmoregulation and salinity tolerance.

Siderophore production is a common PGP trait produced by bacteria [54]. Generally, plant growth-promoting bacteria facilitate nutrient uptake by plants through the production of siderophores in order to sequester iron from the surrounding environments. Halotolerant PGPB that are able to produce siderophores also improve plant health by depleting metals from the rhizosphere, affecting the growth of pathogens, and through the production of antimicrobial compounds [54]. Multiple studies concluded that plant inoculation with siderophore-producing PGPB mitigates salinity effects in plants [55,56,57]. Indeed, several mechanisms to combat oxidative stress include iron ions, such as the antioxidant enzymes catalase, ascorbate peroxidase or iron-superoxide dismutase. In the present study, siderophore production was observed in all strains under control conditions, and exposure to salinity increased siderophore production, significantly in 94% of strains. 

Due to the importance of phosphorus (P) in plant development, growth, photosynthesis, and cell energy (among other key plant functions), the deficiency and unavailability of P in soils severely affects plant metabolism, and subsequently, crop yield. Therefore, PGPB that are able to convert the unavailable forms of phosphorus to a form easily assimilated by plants are important to assure plant vital functions and promote growth and yield [58]. In fact, it is known that phosphorus unavailability is increased by salinity, and an alternative to make phosphorus available for uptake and to increase the salt tolerance of crops is to introduce salt-tolerant phosphate solubilizing bacteria that could supply adequate phosphorus to plants through mobilization of unavailable P forms in the soil [59]. Increased P availability is not only important for cell energy and cell ion homeostasis, but also for regulating cell metabolism under stress conditions, as enzyme phosphorylation is one of the most common ways to regulate enzymatic activity and metabolic pathways. Results evidenced that under control conditions, 83% of strains were able to moderately solubilize phosphate. Exposure to salinity increased phosphate solubilization in 28% of them (11% became high P solubilizers under salt stress). 

Alginate is an extracellular polymeric substance, that is proven to help maintain membrane integrity and cell hydration, for it can hold several times its weight in water [60]. Therefore, it helps bacterial cells to tolerate osmotic stress in water-limited environments [60,61,62]. The accumulation of alginate in bacteria exposed to salt stress is virtually unexplored, emphasizing the importance of the present study for identifying its role in bacterial halotolerance and its influence on plant tolerance to salinity when inoculated with alginate-producing bacteria. Within the presented results, it was observed that all strains were able to produce alginate, with its synthesis enhanced by the exposure to NaCl in most strains in the medium and intracellularly. 

In the present study, salinity led to increased protein carbonylation and reduced lipid peroxidation in most strains, evidencing that proteins were more affected by salinity. It is observed that the activity of CAT and SOD was reduced in most strains exposed to salt stress, contrary to data reported by other authors [63]. On the other hand, GST activity was positively influenced by exposure to salinity, increasing in most strains, and highlighting the crucial role of GST as a detoxification enzyme against salt stress [63]. The higher biochemical changes induced by salinity were observed in proline and protein content, for it significantly increased in most strains. Taking into consideration the osmoregulation and protective roles of proline we are led to think that proline played a crucial role in bacterial osmoadaptation [56,64] and since the proteins determined were the soluble fraction, which are mainly enzymes, it appears that cell metabolism is changed by salinity, with the induction of new or reinforcing existent pathways to respond to salinity.

Salinity is the most important abiotic stress, with detrimental effects on plant growth for it reduces soil osmotic potential, causes nutrient imbalance and the accumulation of Na and Cl ions to interferes with cell metabolism [65]. In the present study salt affected non-inoculated plants, as reported by other authors [66,67]. Inoculation was able to partially alleviate the salt effect, with some strains promoting plant growth, more noticeably the roots (up to 30%). 

Chlorophyll content has a crucial role in photosynthesis, and exposure to salinity was proved to negatively affect photosynthetic pigments, depending on the level of salt stress [51,66,67]. Some studies reported that inoculation with PGPB attenuates salt stress effects on plant chlorophyll levels and that some halotolerant bacteria are able to increase chlorophyll content [67,68]. In the present study, salinity caused the increase in chlorophyll a but an overall reduction in chlorophyll b and carotenoids in non-inoculated plants and in plants inoculated with most strains, yet two strains (A and S) were able to alleviate the negative impact of salinity on photosynthetic pigments. However, it should be noted that the photosynthetic pigments of the maize variety used in this study were higher (by two-fold) than the values reported in other studies [67,68,69]. Therefore, the production of photosynthetic pigments must have been maintained above the capacity of other maize varieties and in a sufficient quantity to support the energy expenditure necessary to combat salinity and support growth. 

Plant mitochondria are crucial for abiotic stress adaptation, for they produce ATP and reducing power to scavenge excess ROS. The activity of the electron transport system in mitochondria can be measured, providing information about plant metabolic activity and respiratory potential [70,71]. Information related to ETS activity in plants exposed to salinity is poorly explored, but Germ et al. [70] described a decrease in ETS activity in abiotically stressed plants. In our study salinity and inoculation decreased ETS activity. Salinity reduces plant growth, thus the energy used in the processes involved in cell division is lower. It seems that the higher energy expenditure to trigger mechanisms minimizing the negative impact of salinity on plants was lower than the energy demand for higher growth and ETS activity was overall lower. 

Most soluble proteins have catalytic activity and are directly involved in metabolic adjustment, playing a crucial role in plant stress response [64]. The activity of ROS-scavenging enzymes (entirely or mostly with a proteic nature) is crucial for the tolerance of plants to salt [72]. In fact, many studies showed increased protein content in salt-stressed plants [50,56,72]. In the present study, salinity slightly increased the protein content in non-inoculated plants, but inoculation with A, F, and Q strains increased the protein content by more than 50%, reaching amounts of 160 mg protein/g FW, considerably higher than the values recorded by El-Esawi et al. [72]. 

As a first line of defense against ROS, plants evolved efficient antioxidant defense systems comprising enzymes such as CAT and SOD that play a crucial role against oxidative damage [22,51,68]. Indeed, salinity increases reactive oxygen species in cells, which are effectively scavenged by these enzymes. Some studies reported an increase in the activity of antioxidant enzymes such as CAT and SOD, with increases in NaCl concentration [44,73,74]. On the contrary, our study showed an overall decrease in CAT activity in inoculated plants both in non-saline and saline conditions, with the exception of plants inoculated with the T strain (110% increase). Salinity significantly decreased SOD activity in non-inoculated plants and in plants inoculated with the A strain; two strains (G and R) increased SOD activity in salt-stressed plants. In light of the results obtained, it seems that plants were not able to trigger the antioxidant systems and in that case, the damage will be overcome; bacterial inoculation reduced the toxicity induced by NaCl on cells and there was no need to induce the antioxidant response since the redox status of cells was not significantly changed.

MDA, a lipid peroxidation product, is an indicator of the cellular oxidative damage caused by ROS, which was reported to accumulate in plant tissues when plants are exposed to salt stress [45]. In the present study, inoculation reduced LPO in non-saline conditions (D, G, Q, R and T), but under salt stress, LPO content increased in inoculated plants. Similarly, several studies reported LPO increases in plants under salt stress [18,45,68,69]. 

Protein carbonylation is used as a biomarker of oxidative stress in proteins, and a sensitive indicator of damage, since carbonylated proteins are catabolized slowly [75]. Osmotic stress caused by drought and salinity results in higher protein carbonylation in plants [75,76]. In this study, the quantification of protein damage in maize plants under salt stress evidenced that inoculation with PGPB contributed to stress alleviation, a virtually unreported effect. Inoculation with some strains reduced protein damage under non-saline (D, F, G, Q, R, S and T), and saline (A, D, F and G) conditions, proving that inoculation can reduce the damaging effects of salinity. This result has biological relevance since protein protection from damage reduces the impact on enzyme activity and on metabolic pathways and allows the cell to adapt more efficiently to the intracellular conditions imposed by salt stress. The reduction in protein levels may partially explain the non-increase in the antioxidant activity of cells in salt-exposed plants. 

Besides the increase in ROS, Na^+^ and Cl^−^ ions also have osmotic effects, being essential for cells to adjust osmotically. Soluble sugars play an important role in osmoregulation. In addition to the osmotic effect, soluble sugars also act indirectly during plant growth and development by regulating carbohydrate metabolism under salt stress [72,77]. Several studies reported the accumulation of soluble sugars in plants under salt stress, and some presented the positive contribution of PGPB in soluble sugars accumulation [45,50,72,78,79]. The results obtained in our study contradict this trend with salt-stressed non-inoculated plants evidencing lower levels of soluble sugars, but with some strains (D, G, Q and T) reducing this effect. 

Salinity also promotes the accumulation of proline, which plays a crucial role in the adaptation of cells to saline conditions [80]. Proline acts as an osmolyte regulating cell water potential, helps in the detoxification of reactive oxygen species, protects membrane integrity and acts as a molecular chaperone, therefore, assuring protein structure stabilization [81]. The accumulation of proline in salt-stressed plants is documented [82,83,84]. Our study validates these observations with significant increases in proline in salt-stressed plants both inoculated and non-inoculated. Inoculation with some strains (D, Q and R) further increased proline levels, reaching 200 mg proline/g plant FW (R). This proline concentration is substantially higher than the levels reported in other studies on maize plants under salt stress and inoculated with PGPB [46,82] and evidences the extra protection conferred by inoculation with these three strains to plants exposed to salinity.

## 5. Conclusions

The use of soil bacteria from salt-affected areas to increase the tolerance of plants to salinity proved to be a successful strategy. After the isolation of bacteria from the roots of natural plants growing in an arid and sea-influenced region, the Sal Island in Cape Verde, the halotolerance of strains and the effect of salinity on the biochemical status of bacteria was studied, with the aim to increase the knowledge about salinity effect on bacteria biochemistry. All strains were halotolerant, with some being extremely tolerant to NaCl. Proline and protein content were the main biochemical changes induced by salinity in bacteria, evidencing that proline played a crucial role in bacterial osmoadaptation and protection from ion toxicity and that cell metabolism was changed by salinity. The ability of bacteria to promote plant growth in saline conditions was also determined. Most PGP traits were strongly enhanced by exposure to salt stress, contradicting previous results and more importantly, supporting the use of bioinoculants to promote plant growth under saline conditions. The effect of bacterial inoculation on salt-stressed plants was also evaluated as a way to increase knowledge on the effects of PGPB inoculation on plant tolerance to salinity. The root growth of salt-stressed plants was promoted by the ability of strains to produce phytohormones, siderophores and phosphate solubilization. The increase in osmolyte concentration, mainly proline, had a positive effect on plant tolerance to salinity. 

This study evidences relevant results for environmental and agricultural contexts since inoculation with bacterial halotolerant strains can be of major value to reduce the impact of salinity on crops to reclaim salinized land and contribute to food security.

## Data Availability

Data are contained within the article and Supplementary Material.

## Supplementary Materials

The following are available online at https://www.mdpi.com/article/10.3390/antiox12020488/s1, Table S1: Strains characterization: bacterial code, genera, accession number and which plant were isolated from; Table S2: Plant growth-promoting abilities of bacteria isolated from Cape Verde. Determination of the percentage of NaCl that inhibits 50% of bacterial growth (IC_50_ NaCl); Table S3: Results obtained for biochemical parameters of bacterial strains grown in osmotic stress (% NaCl inhibiting growth 50%) and control (no NaCl added); Table S4: Maize plants grown for 7 days in non-saline and saline conditions. Morphometric parameters (fresh weight and length of plants) and variation of photosynthetic pigments in inoculated (A, D, F, G, Q, R, S, T) and non-inoculated (control—Ctl) plants; Table S5: Maize plants grown for 7 days in non-saline and saline conditions. Biochemical parameters evaluated in inoculated (A, D, F, G, Q, R, S, T strains) and non-inoculated (control—Ctl) plants; Table S6: PCO Pearson-correlation values of Figure 3 and Figure 5.
