# Supplementary material for: Application of Plant Growth-Promoting Bacteria from Cape Verde to Increase Maize Tolerance to Salinity
Source: Antioxidants (Basel). 2023 Feb 15;12(2):488. doi: 10.3390/antiox12020488 (PMC9952022; doi:10.3390/antiox12020488)
Supplement: Supplementary file 1 [file antioxidants-12-00488-s001.zip › Supplementary tables.pdf]

Table S1: Strains characterization: bacterial code, genera, accession number and which plant were isolated from.

**Code**	**Species**	**Accession number**	**Isolated from**
A	*Pantoea sp.*	OM985627	*Acacia albida*
B	*Klebsiella sp.*	OM985630	*Acacia albida*
D	*Pseudomonas sp.*	OM985635	*Acacia albida*
E	*Pseudomonas sp.*	OM985638	*Acacia albida*
F	*Acinetobacter sp.*	OM985640	*Acacia albida*
G	*Stenotrophomonas sp.*	OM985622	*Acacia albida*
H	*Enterobacter sp.*	OM985625	*Acacia albida*
J	*Enterobacter sp.*	OM985623	*Amaranthus viridis*
K	*Pantoea sp.*	OM985626	*Amaranthus viridis*
L	*Pseudomonas sp.*	OM985629	*Amaranthus viridis*
M	*Rhizobium sp.*	OM985632	*Acacia albida*
N	*Paenarthrobacter sp.*	OM985634	*Acacia albida*
O	*Ochrobactrum sp.*	OM985637	*Acacia albida*
Q	*Pseudomonas sp.*	OM985621	*Acacia albida*
R	*Rhizobium sp.*	OM985624	*Acacia albida*
S	*Stenotrophomonas sp.*	OM985631	*Acacia albida*
T	*Pseudomonas sp.*	OM985633	*Acacia albida*
U	*Enterobacter sp.*	OM985636	*Acacia albida*

Table S2: Plant growth-promoting abilities of bacteria isolated from Cape Verde. Determination of the percentage of NaCl that inhibits 50% of bacterial growth (IC50 NaCl). Indole acetic acid (IAA) and alginate production were expressed in µg/mL/DO. Siderophore production was expressed in percent siderophore units per optical density (PSU/DO). Phosphate solubilization was expressed by the ratio between halo and colony diameter. Values are means of three replicates + standard deviation. Statistical analysis was performed relatively to non-inoculated control. Significant differences were considered for $p \leq 0.05$ and were identified with values in bold and single asterisks (for $p \leq 0.05$) and double asterisks (for $p \leq 0.01$).

		Siderophores (PSU/OD)		IAA (µg/ml/OD)		Phosphate solubilization (ratio)		Total alginate (µg/ml/OD)		Alginate - medium (µg/ml/OD)		Extracellular alginate (µg/ml/OD)		Intracellular alginate (µg/ml/OD)	
	IC50 NaCl	control	salinity	control	salinity	control	salinity	control	salinity	control	salinity	control	salinity	control	salinity
**A**	5	22.04 ± 2.73	**81.69 ± 4.57****	4.23 ± 0.48	**6.49 ± 0.33****	1.18 ± 0.09	**1.75 ± 0.25***	26.32 ± 3.57	**34.33 ± 2.07****	12.47 ± 0.73	**17.10 ± 0.41****	6.78 ± 0.29	6.17 ± 0.93	7.07 ± 2.63	**11.06 ± 1.32****
**B**	5	13.96 ± 4.39	**43.44 ± 2.06****	5.60 ± 0.33	5.55 ± 0.59	1.25 ± 0.07	1.67 ± 0.33	23.06 ± 0.55	**27.11 ± 2.02****	13.02 ± 0.23	**13.52 ± 0.10****	6.73 ± 0.35	**6.35 ± 0.13***	3.31 ± 0.52	**7.24 ± 1.98****
**D**	4.5	23.43 ± 4.25	**39.84 ± 5.57***	4.44 ± 0.39	**5.69 ± 0.33****	1.25 ± 0.14	**1.75 ± 0.25***	28.68 ± 1.09	29.24 ± 1.43	15.33 ± 0.63	14.59 ± 0.69	9.55 ± 0.33	**7.60 ± 0.32****	3.80 ± 0.43	**7.05 ± 0.89****
**E**	5	18.94 ± 3.14	**82.06 ± 11.65****	4.76 ± 0.43	**10.27 ± 0.74****	-	-	28.37 ± 2.31	**47.03 ± 2.65****	12.84 ± 0.67	**24.37 ± 0.78****	10.58 ± 0.24	**13.70 ± 0.65****	4.95 ± 1.91	**8.97 ± 2.50***
**F**	5.5	32.23 ± 5.45	**50.59 ± 4.12****	3.72 ± 0.52	**6.59 ± 0.38****	1.71 ± 0.21	1.83 ± 0.17	26.61 ± 0.62	**30.64 ± 0.77****	14.05 ± 0.21	**15.92 ± 0.42****	8.61 ± 0.43	**6.96 ± 0.27****	3.95 ± 0.39	**7.76 ± 0.94****
**G**	4.5	16.12 ± 2.29	**45.49 ± 4.61****	4.36 ± 0.45	**5.90 ± 0.67****	1.03 ± 0.09	**2.06 ± 0.59***	15.89 ± 1.34	**26.65 ± 0.92****	4.95 ± 1.19	**10.36 ± 0.27****	6.75 ± 0.18	**5.17 ± 0.30****	4.20 ± 0.33	**11.13 ± 1.18****
**H**	5	13.90 ± 8.64	**48.94 ± 3.45****	4.75 ± 0.46	**7.03 ± 0.37****	1.97 ± 0.19	1.50 ± 0.33	19.53 ± 1.03	**26.96 ± 2.43****	6.13 ± 0.41	**11.35 ± 0.57****	9.66 ± 0.22	**4.71 ± 0.19****	3.73 ± 0.69	**10.90 ± 2.31****
**J**	5.5	25.83 ± 1.80	**54.72 ± 7.84****	9.62 ± 0.83	**7.40 ± 0.74****	1.88 ± 0.27	1.43 ± 0.21	27.65 ± 3.09	**32.47 ± 2.93***	7.37 ± 0.64	**11.32 ± 0.07****	10.89 ± 2.84	10.08 ± 3.01	9.39 ± 1.35	**11.07 ± 0.66***
**K**	6	22.05 ± 7.83	52.42 ± 17.32	9.87 ± 1.23	**8.58 ± 0.30***	1.32 ± 0.12	1.49 ± 0.10	34.20 ± 0.74	35.66 ± 2.29	12.24 ± 0.76	**15.91 ± 1.05****	13.31 ± 0.44	12.57 ± 0.79	8.65 ± 0.43	**7.18 ± 0.63***
**L**	3	57.54 ± 4.89	**65.91 ± 4.10***	4.68 ± 1.33	**9.95 ± 1.03****	1.40 ± 0.07	**1.67 ± 0.04***	31.69 ± 4.77	**41.23 ± 1.67****	3.63 ± 0.98	**8.27 ± 0.68****	11.44 ± 0.89	**15.23 ± 1.24****	16.62 ± 3.38	17.73 ± 2.27
**M**	6	22.54 ± 5.76	**101.84 ± 47.75****	4.17 ± 0.52	**13.59 ± 12.26****	1.08 ± 0.14	-	25.18 ± 1.99	**32.01 ± 2.14****	3.34 ± 0.67	**8.46 ± 0.13****	10.78 ± 1.10	**13.33 ± 0.34****	11.06 ± 2.91	10.23 ± 1.97
**N**	2.25	49.94 ± 4.64	**283.70 ± 38.37****	5.71 ± 0.80	29.56 ± 4.73	-	-	11.44 ± 2.49	**36.12 ± 7.51****	1.73 ± 0.48	**9.49 ± 4.63****	5.70 ± 0.72	**16.23 ± 7.51****	4.01 ± 2.38	5.44 ± 3.43
**O**	5	17.77 ± 0.96	**44.29 ± 8.05****	6.15 ± 0.80	14.36 ± 10.73	1.94 ± 0.14	**1.45 ± 0.05***	19.81 ± 1.66	**24.06 ± 0.67****	2.28 ± 0.23	**4.81 ± 0.20****	10.56 ± 0.72	**9.59 ± 0.31***	6.97 ± 1.34	**9.67 ± 0.70****
**Q**	5	15.77 ± 5.69	**59.24 ± 10.88****	6.39 ± 0.88	**9.37 ± 1.02****	1.04 ± 0.03	**1.46 ± 0.26***	22.80 ± 4.28	26.07 ± 3.84	5.55 ± 3.33	8.08 ± 3.21	10.39 ± 0.31	**7.79 ± 1.27****	6.86 ± 1.18	**10.19 ± 1.72****
**R**	6	23.29 ± 8.10	**75.97 ± 9.09****	4.67 ± 0.34	**7.10 ± 0.13****	1.15 ± 0.03	-	23.40 ± 1.44	27.08 ± 4.54	3.60 ± 1.93	4.33 ± 1.62	11.11 ± 0.87	11.01 ± 1.54	8.70 ± 1.89	**11.74 ± 1.47***
**S**	3	51.83 ± 5.77	**208.36 ± 49.45****	7.23 ± 0.41	**15.47 ± 1.14****	-	-	43.49 ± 3.03	36.80 ± 12.14	6.87 ± 3.56	10.67 ± 6.02	18.29 ± 1.19	18.72 ± 4.10	18.34 ± 4.89	**7.41 ± 2.55****
**T**	5	12.04 ± 3.77	**48.94 ± 3.24****	4.34 ± 0.61	**5.45 ± 0.40***	1.38 ± 0.24	2.33 ± 0.58	23.06 ± 1.80	22.47 ± 1.78	2.74 ± 1.38	3.69 ± 1.68	10.50 ± 0.78	**8.14 ± 1.19****	9.81 ± 0.67	**10.64 ± 0.31***
**U**	5.5	13.91 ± 1.32	**46.05 ± 1.46****	3.80 ± 0.57	**5.96 ± 0.44****	1.88 ± 0.17	1.56 ± 0.24	30.22 ± 2.88	**24.21 ± 2.41****	8.98 ± 2.51	8.86 ± 2.47	10.69 ± 1.21	**7.18 ± 1.99****	10.55 ± 0.50	**8.17 ± 0.90****

Table S3: Results obtained for biochemical parameters of bacterial strains grown in osmotic stress (% NaCl inhibiting growth 50%) and control (no NaCl added). Bacterial strains: *Pantoea spp*. (A); *Klebsiella spp*. (B); *Pseudomonas spp*. (D); *Pseudomonas spp*. (E); *Acinetobacter spp*. (F); *Stenotrophomonas spp*. (G); *Enterobacter spp*. (H); *Enterobacter spp*. (J); *Pantoea spp*. (K); *Pseudomonas spp*. (L); *Rhizobium spp*. (M); *Paenarthrobacter spp*. (N); *Ochrobactrum spp*. (O); *Pseudomonas spp*. (Q); *Rhizobium spp*. (R); *Stenotrophomonas spp*. (S); *Pseudomonas spp*. (T); *Enterobacter spp*. (U). Values are means of three replicates + standard deviation. Statistical analysis was performed relatively to non-inoculated control. Significant differences were considered for $p \leq 0.05$ and were identified with values in bold and single asterisks (for $p \leq 0.05$) and double asterisks (for $p \leq 0.01$).

	LPO (pmoles MDA/M cells)		SOD (µU/M cells)		CAT (µU/M cells)		PROLINE (µg/M cells)		GST (µU/M cells)		PC (mmol/M cells)		PROT (µg prot/M cells)	
	control	salinity	control	salinity	control	salinity	control	salinity	control	salinity	control	salinity	control	salinity
A	2.14 ± 0.23	**3.32 ± 0.77****	125.15 ± 6.38	139.64 ± 18.75	2.77 ± 0.53	2.78 ± 0.87	11.39 ± 2.64	13.47 ± 3.29	3.59 ± 0.19	3.31 ± 0.72	0.70 ± 0.03	0.55 ± 0.12	0.23 ± 0.04	0.48 ± 0.11
B	2.91 ± 0.53	**1.51 ± 0.52***	117.56 ± 8.85	107.38 ± 15.59	2.53 ± 0.11	1.76 ±0.46	5.03 ± 2.22	6.86 ± 1.63	3.06 ± 0.59	4.07 ± 1.05	0.52 ± 0.05	**0.32 ± 0.07***	0.27 ± 0.09	0.32 ± 0.07
D	3.02 ± 0.40	2.29 ± 0.67	125.46 ± 9.36	125.23 ± 19.81	2.61 ± 0.52	2.18 ± 0.91	2.03 ± 0.38	**3.04 ± 0.44***	3.11 ± 0.67	5.53 ± 1.55	0.49 ± 0.09	0.70 ± 0.14	0.30 ± 0.11	0.23 ± 0.06
E	3.37 ± 0.43	3.54 ± 1.13	135.57 ± 13.82	161.78 ± 38.10	1.83 ± 0.20	**3.17 ± 0.19***	6.74 ± 2.20	12.45 ± 3.89	5.00 ± 0.76	**3.50 ± 0.39***	0.53 ± 0.05	0.57 ± 0.07	0.23 ± 0.03	**0.42 ± 0.09***
F	1.75 ± 0.32	2.89 ± 0.71	124.46 ± 23.59	155.21 ± 38.27	2.45 ± 0.77	2.33 ± 0.60	2.43 ± 0.49	2.30 ± 0.58	2.98 ± 0.17	3.52 ± 0.55	0.30 ± 0.06	0.40 ± 0.07	0.26 ± 0.05	0.24 ± 0.07
G	1.89 ± 0.47	2.76 ± 0.38	130.53 ± 3.78	**114.59 ± 3.99****	2.98 ± 0.69	1.69 ± 0.82	1.73 ± 0.46	**9.86 ± 1.44****	3.22 ± 0.31	3.86 ± 1.12	0.42 ± 0.07	0.51 ± 0.10	0.19 ± 0.07	0.26 ± 0.04
H	2.94 ± 0.33	**1.42 ± 0.12****	117.58 ± 14.85	93.66 ± 30.29	9.62 ± 0.88	**3.35 ± 1.27***	1.76 ± 0.51	3.75 ± 1.41	3.69 ± 0.58	3.08 ± 0.20	0.35 ± 0.01	**0.28 ± 0.04***	0.16 ± 0.04	0.23 ± 0.07
J	1.83 ± 0.36	1.77 ± 0.45	121.58 ± 1.24	**96.20 ± 7.62****	11.19 ± 1.46	**1.44 ± 0.44****	3.52 ± 0.47	**6.18 ± 1.41***	3.87 ± 0.46	4.25 ± 0.59	0.50 ± 0.05	**0.38 ± 0.05***	0.29 ± 0.05	0.22 ± 0.07
K	4.33 ± 1.13	2.66 ± 0.44	122.65 ± 17.79	63.85 ± 9.66	1.66 ± 0.63	1.98 ± 0.37	1.16 ± 0.48	**4.85 ± 0.93****	2.74 ± 0.50	**11.10 ± 0.49****	0.38 ± 0.07	**1.23 ± 0.12****	0.25 ± 0.05	0.33 ± 0.05
L	2.96 ± 0.94	2.40 ± 0.52	145.96 ± 7.00	**83.80 ± 14.06****	1.29 ± 0.25	**2.47 ± 0.19****	0.35 ± 0.21	**4.97 ± 1.79***	5.51 ± 0.50	5.23 ± 0.07	0.71 ± 0.07	**0.52 ± 0.05****	0.30 ± 0.07	**1.07 ± 0.20****
M	1.49 ± 0.48	2.18 ± 0.28	140.51 ± 7.57	**52.12 ± 13.65****	9.76 ± 4.53	**1.37 ± 0.06***	1.92 ± 0.13	**3.85 ± 0.74***	6.51 ± 1.44	4.82 ± 0.65	0.55 ± 0.12	0.51 ± 0.02	0.24 ± 0.04	0.19 ± 0.01
N	9.18 ± 1.55	7.76 ± 1.66	132.19 ± 0.53	**54.76 ± 28.52****	2.34 ± 0.45	2.92 ± 1.91	3.03 ± 0.37	2.55 ± 1.14	3.83 ± 0.78	6.48 ± 1.59	0.70 ± 0.12	0.69 ± 0.10	0.22 ± 0.04	0.88 ± 0.41
O	2.70 ± 0.36	**1.00 ± 0.12****	136.43 ± 7.80	**50.78 ± 5.77****	17.93 ± 3.26	**1.48 ± 0.03****	1.10 ± 0.48	**3.02 ± 0.39****	2.88 ± 0.33	3.53 ± 0.61	0.35 ± 0.09	0.43 ± 0.09	0.27 ± 0.02	0.40 ± 0.09
Q	3.05 ± 0.80	**0.76 ± 0.26****	146.52 ± 7.58	108.60 ± 32.85	14.97 ± 7.31	**1.54 ± 0.60***	0.41 ± 0.13	**16.26 ± 4.00****	3.23 ± 0.14	**4.97 ± 0.29***	0.33 ± 0.03	**0.68 ± 0.08****	0.14 ± 0.01	**0.42 ± 0.02****
R	2.04 ± 0.18	**0.85 ± 0.11****	142.10 ± 10.50	**64.33 ± 10.41****	3.43 ± 0.24	**1.15 ± 0.26****	2.67 ± 0.76	**12.89 ± 1.96****	3.12 ± 0.30	3.51 ± 0.37	0.29 ± 0.03	**0.42 ± 0.002****	0.14 ± 0.03	**0.25 ± 0.02****
S	2.43 ± 0.15	**0.03 ± 0.01****	133.85 ± 12.69	**109.50 ± 5.92***	2.11 ± 0.67	2.06 ± 0.41	9.19 ± 2.43	9.34 ± 0.48	3.38 ± 0.20	**10.16 ± 1.84****	0.33 ± 0.04	**0.84 ± 0.12****	0.29 ± 0.03	0.32 ± 0.09
T	3.01 ± 0.39	**4.31 ± 0.64***	147.21 ± 5.53	**57.74 ± 21.68****	2.79 ± 1.04	1.73 ± 0.31	3.30 ± 0.50	**9.98 ± 2.01****	3.46 ± 0.64	3.87 ± 0.13	0.32 ± 0.04	**0.45 ± 0.07***	0.35 ± 0.15	0.42 ± 0.12
U	3.06 ± 0.49	**1.74 ± 0.48***	139.07 ± 11.08	**86.22 ± 21.31***	1.41 ± 0.33	1.73 ± 0.29	0.97 ± 0.40	**4.56 ± 1.26****	3.96 ± 0.59	4.66 ± 0.65	0.30 ± 0.04	**0.48 ± 0.04****	0.08 ± 0.01	**0.53 ± 0.18***

Table S4: Maize plants grown for 7 days in non-saline and saline conditions. Morphometric parameters (fresh weight and length of plants) and variation of photosynthetic pigments in inoculated (A, D, F, G, Q, R, S, T) and non-inoculated (control – Ctl) plants. Values are means of three replicates + standard deviation. Statistical analysis was performed relatively to non-inoculated control. Significant differences were considered for $p \leq 0.05$ and were identified with values in bold with single asterisks (for $p \leq 0.05$) and double asterisks (for $p \leq 0.01$). Bacterial strains: *Pantoea spp.* (A); *Pseudomonas spp.* (D); *Acinetobacter spp.* (F); *Stenotrophomonas spp.* (G); *Pseudomonas spp.* (Q); *Rhizobium spp.*(R); *Stenotrophomonas spp.* (S); *Pseudomonas spp.* (T).

	Shoot Fresh Weight (g)		Root Fresh Weight (g)		Shoot length (cm)		Root length (cm)		Chl a (µg/g FW)		Chl b (µg/g FW)		Carotenoids (µg/g FW)	
	control	salinity	control	salinity	control	salinity	control	salinity	control	salinity	control	salinity	control	salinity
**Ctl**	0.32 ± 0.02	**0.23 ± 0.02***	1.91 ± 0.15	**1.32 ± 0.31***	9.00 ± 0.33	**7.36 ± 0.71***	8.08 ± 8.39	7.44 ± 0.10	378.78 ± 108.73	560.17 ± 75.80	196.83 ± 61.22	148.20 ± 17.29	152.35 ± 34.82	120.59 ± 22.82
**A**	0.32 ± 0.04	**0.24 ± 0.03***	**1.49 ± 0.06***	1.67 ± 0.14	9.33 ± 1.15	9.42 ± 0.42	8.33 ± 0.33	8.61 ± 1.13	331.00 ± 133.64	656.75 ± 241.97	165.56 ± 69.71	176.71 ± 64.19	146.99 ± 51.16	135.82 ± 33.09
**D**	0.35 ± 0.03	**0.18 ± 0.04***	1.65 ± 0.22	**1.44 ± 0.05***	8.08 ± 0.65	**7.78 ± 0.63***	7.78 ± 0.77	9.00 ± 0.67	274.78 ± 118.33	481.57 ± 37.94	134.10 ± 52.64	138.66 ± 7.30	119.49 ± 47.64	98.44 ± 10.22
**F**	0.32 ± 0.06	0.26 ± 0.05	1.91 ± 0.26	1.69 ± 0.34	8.61 ± 0.67	8.44 ± 0.84	8.44 ± 0.69	8.59 ± 0.56	275.59 ± 116.35	429.33 ± 120.05	135.92 ± 50.37	119.86 ± 22.98	113.45 ± 45.52	**87.74 ± 23.57***
**G**	0.32 ± 0.05	**0.23 ± 0.05***	1.60 ± 0.30	1.46 ± 0.24	8.89 ± 0.51	7.91 ± 0.69	7.67 ± 0.58	8.04 ± 0.60	302.32 ± 87.19	355.87 ± 120.86	147.16 ± 40.40	105.00 ± 28.96	135.81 ± 29.09	**74.29 ± 24.24***
**Q**	0.34 ± 0.06	**0.21 ± 0.03***	**1.46 ± 0.15***	1.67 ± 0.13	8.83 ± 0.67	**7.06 ± 0.86***	8.33 ± 1.01	8.28 ± 0.54	372.24 ± 49.19	477.58 ± 283.99	176.54 ± 27.82	138.33 ± 72.37	157.37 ± 20.30	106.33 ± 56.88
**R**	0.33 ± 0.05	**0.18 ± 0.01****	1.55 ± 0.31	1.56 ± 0.26	8.39 ± 0.79	**6.56 ± 0.63***	7.33 ± 0.44	**9.56 ± 0.51***	286.93 ± 95.30	508.48 ± 87.65	138.09 ± 40.96	147.6 ± 16.01	127.27 ± 31.73	106.42 ± 15.36
**S**	0.33 ± 0.06	**0.24 ± 0.04***	1.95 ± 0.15	1.60 ± 0.36	9.17 ± 0.76	8.61 ± 1.39	8.22 ± 0.51	8.83 ± 0.50	411.88 ± 40.07	**593.84 ± 92.29***	204.15 ± 23.01	165.95 ± 12.68	179.00 ± 27.84	121.96 ± 27.54
**T**	0.31 ± 0.05	**0.24 ± 0.05***	**1.42 ± 0.19***	1.80 ± 0.30	8.17 ± 0.93	7.78 ± 1.30	7.83 ± 0.44	**7.11 ± 0.19***	**220.00 ± 60.98***	484.53 ± 105.61	**111.52 ± 29.50***	139.83 ± 24.60	**97.33 ± 20.92***	103.71 ± 23.68

Table S5: Maize plants grown for 7 days in non-saline and saline conditions. Biochemical parameters evaluated in inoculated (A, D, F, G, Q, R, S, T strains) and non-inoculated (control – Ctl) plants. Values are means of three replicates + standard deviation. Statistical analysis was performed relatively to non-inoculated (control – Ctl) non-saline plants. Significant differences were considered for $p \leq 0.05$ and were identified with values in bold and single asterisks (for $p \leq 0.05$) and double asterisks (for $p \leq 0.01$). Bacterial strains: *Pantoea spp.* (A); *Pseudomonas spp.* (D); *Acinetobacter spp.* (F); *Stenotrophomonas spp.* (G); *Pseudomonas spp.* (Q); *Rhizobium spp.*(R); *Stenotrophomonas spp.* (S); *Pseudomonas spp.* (T).

	**LPO (nmoles MDA/g FW)**		**SOD (mU/g FW)**		**CAT (mU/g FW)**		**PROLINE (mg/g FW)**		**ETS (nmol/min * g FW)**		**PC (µmol/g FW)**		**PROT (mg prot/g FW)**		**SOLUBLE SUGARS (mg/g FW)**	
	**control**	**salinity**	**control**	**salinity**	**control**	**salinity**	**control**	**salinity**	**control**	**salinity**	**control**	**salinity**	**control**	**salinity**	**control**	**salinity**
**Ctl**	3.56 ± 0.93	3.85 ± 0.99	0.29 ± 0.04	**0.08 ± 0.02***	35.45 ± 7.11	21.57 ± 5.43	68.47 ± 15.14	**137.28 ± 16.16****	21.58 ± 4.15	14.25 ± 3.26	1.28 ± 0.36	**0.54 ± 0.12***	66.62 ± 9.03	76.48 ± 23.40	18.26 ± 4.23	**1.31 ± 0.27***
**A**	5.91 ± 1.38	4.87 ± 0.97	0.33 ± 0.06	**0.09 ± 0.02***	**49.46 ± 3.73***	28.99 ± 2.79	**113.09 ± 18.06***	**106.13 ± 12.61***	16.99 ± 3.38	13.91 ± 2.87	1.83 ± 0.59	**0.57 ± 0.08***	95.61 ± 16.01	124.70 ± 23.40	20.45 ± 1.61	**8.55 ± 1.37***
**D**	3.55 ± 0.57	5.23 ± 0.65	0.34 ± 0.04	0.22 ± 0.07	26.47 ± 4.02	31.41 ± 7.52	**108.75 ± 10.06***	**180.24 ± 26.30***	16.37 ± 3.70	11.43 ± 1.91	0.84 ± 0.27	0.92 ± 0.20	78.17 ± 16.80	76.30 ± 17.06	17.02 ± 3.45	19.71 ± 4.03
**F**	4.49 ± 0.81	4.15 ± 0.11	0.21 ± 0.05	0.22 ± 0.04	32.36 ± 11.02	24.98 ± 1.61	69.89 ± 22.11	**132.60 ± 2.64***	23.28 ± 3.63	16.39 ± 3.94	1.04 ± 0.15	0.94 ± 0.30	71.11 ± 7.98	**129.98 ± 32.39***	24.41 ± 6.43	**2.94 ± 0.43***
**G**	2.38 ± 0.64	5.04 ± 0.60	**0.18 ± 0.04***	0.42 ± 0.10	23.77 ± 6.67	22.49 ± 2.02	63.99 ± 10.66	**138.84 ± 20.04***	13.74 ± 2.97	12.90 ± 2.12	0.97 ± 0.25	0.86 ± 0.31	73.58 ± 11.84	92.30 ± 16.10	16.98 ± 3.57	11.77 ± 1.18
**Q**	2.59 ± 0.67	**5.38 ± 0.40***	0.21 ± 0.03	0.36 ± 0.04	**22.29 ± 1.09***	26.19 ± 3.61	78.85 ± 12.62	**160.48 ± 22.28***	15.91 ± 2.19	16.00 ± 2.05	**0.63 ± 0.16***	1.71 ± 0.25	95.74 ± 17.65	**161.36 ± 40.02***	**10.55 ± 1.28***	11.60 ± 1.09
**R**	2.34 ± 0.76	**6.10 ± 1.21***	**0.12 ± 0.03***	**0.45 ± 0.07***	**18.64 ± 3.57***	36.03 ± 3.86	71.20 ± 11.97	**198.94 ± 29.12***	17.58 ± 0.77	26.64 ± 5.11	**0.54 ± 0.08***	1.47 ± 0.43	62.63 ± 8.81	77.71 ± 18.32	18.70 ± 1.04	**6.07 ± 1.20***
**S**	4.01 ± 0.95	4.51 ± 0.35	0.21 ± 0.03	0.28 ± 0.05	30.03 ± 5.54	29.71 ± 8.73	92.86 ± 13.24	**125.52 ± 21.76***	23.90 ± 6.05	19.78 ± 2.24	**0.59 ± 0.08***	1.28 ± 0.36	69.36 ± 20.25	69.87 ± 3.73	19.43 ± 4.46	**9.19 ± 0.57***
**T**	3.47 ± 0.36	5.30 ± 1.35	0.34 ± 0.05	0.33 ± 0.06	25.92 ± 1.39	45.74 ± 12.53	95.96 ± 11.75	**130.41 ± 19.31***	19.28 ± 1.86	24.04 ± 2.92	1.22 ± 0.16	1.72 ± 0.31	106.69 ± 38.20	76.60 ± 12.30	24.24 ± 3.75	12.40 ± 1.97

Table S6: PCO Pearson-correlation values of figure 3 and 5.

	**Bacteria (Figure 3)**	**Plants (figure 5)**
**PROT**	0.39	0.53
**Proline**	0.64	0.89
**SOD**	0.71	0.93
**PC**	0.94	0.8
**LPO**	0.76	0.97
**CAT**	0.5	0.76
**GST**	0.96	
**ETS**		0.81
**Sol Sug**		0.85
